# Epidemiological and clinical profile of adult patients with *Blastocystis* sp. infection in Barcelona, Spain

**DOI:** 10.1186/s13071-016-1827-4

**Published:** 2016-10-14

**Authors:** Fernando Salvador, Elena Sulleiro, Adrián Sánchez-Montalvá, Carmen Alonso, Javier Santos, Isabel Fuentes, Israel Molina

**Affiliations:** 1 Department of Infectious Diseases, Vall d’Hebron University Hospital, Universitat Autònoma de Barcelona, PROSICS Barcelona, Barcelona, Spain; 2 Department of Microbiology, Vall d’Hebron University Hospital, PROSICS Barcelona, Barcelona, Spain; 3 Gastroenterology Department, Vall d’ Hebron Research Institute, Digestive Diseases Research Unit, Vall d’Hebron University Hospital, Barcelona, Spain; 4 Department of Medicine, Universitat Autònoma de Barcelona, Centro de Investigación Biomédica en Red de Enfermedades Hepáticas y Digestivas (Ciberehd), Barcelona, Spain; 5 Instituto de Salud Carlos III, National Centre of Microbiology, Madrid, Spain

**Keywords:** *Blastocystis*, Intestinal protozoa, Gastrointestinal symptoms, Metronidazole

## Abstract

**Background:**

*Blastocystis* spp. are among the most frequently observed intestinal parasites in humans. Despite the discovery of *Blastocystis* approximately 100 years ago, limited information is available regarding its pathogenesis, genetic diversity, and available treatment options. The aim of this study was to describe the epidemiological and clinical characteristics of patients with *Blastocystis* sp. infections diagnosed at Vall d’Hebron University Hospital (Barcelona, Spain).

**Methods:**

A retrospective observational study was performed which included all adult patients who attended Vall d’Hebron University Hospital from February 2009 to March 2014 that had *Blastocystis* sp. detected in their stool.

**Results:**

Four hundred eighteen patients were included, the median age was 36 (18–86) years and 236 (56.5 %) were men. Regarding patient symptoms, 234 (56 %) patients were completely asymptomatic, 92 (22 %) patients had symptoms, and 92 (22 %) patients had symptoms that could be attributed to other causes. Of the 92 patients with symptoms not attributable to other etiologies except for *Blastocystis* infection, the most frequent symptoms were diarrhea (61 patients, 66.3 %) and abdominal pain (34 patients, 37 %). Additionally, nine (9.8 %) patients had cutaneous manifestations. Thirty-one (7.4 %) patients received specific treatment for *Blastocystis* infection. The clinical response of treated patients was varied. Five patients experienced complete resolution of symptoms, 12 patients reported improvement of clinical symptoms, eight patients described no clinical improvement, and information was unavailable for six patients.

**Conclusions:**

*Blastocystis* infection was detected in 418 patients, most of them foreign-born. Although the vast majority of patients were asymptomatic, 22 % of patients had gastrointestinal symptoms or cutaneous manifestations in the absence of other causes. Despite the scarce information available, given the safety of antiparasitic treatment, and the percentage of patients who experienced resolution or improvement of symptoms, treatment should be considered in patients with chronic symptoms.

## Background

Parasitic diseases are amongst the most important causes of morbidity and mortality worldwide, and it is estimated that nearly 340 parasite species infect humans. Of these species intestinal protozoa are the most prevalent [1]. The pathogenicity of several intestinal protozoa is well established, and their presence is usually associated with diarrhea and other intestinal manifestations (viz. *Giardia duodenalis*, *Entamoeba histolytica*, *Cryptosporidium* spp., *Cystoisospora belli*). Some protozoa cause significant morbidity and sometimes mortality, especially among immunosuppressed populations [2].

In contrast, the role of other intestinal protozoa in the development of human disease is not well established, and their presence in both symptomatic and asymptomatic patients is difficult to explain [1]. *Blastocystis* spp. are among the most frequently observed intestinal parasites in humans. Despite its description approximately 100 years ago, little information is available regarding their pathogenesis, genetic diversity, host range and available treatment options [3]. *Blastocystis* has a worldwide distribution, with higher prevalences reported in developing countries. The parasite is transmitted by the fecal-oral route, and animal reservoirs include chickens, rats and pigs [4]. Seventeen subtypes (STs) have been described based on analyses of the small subunit rRNA (SSU rRNA) gene. Only STs 1–9 have been found in humans, ST-3 being the most prevalent in epidemiological studies [5, 6].

Clinical manifestations associated with *Blastocystis* spp. carriage include diarrhea, abdominal pain, vomiting and other gastrointestinal symptoms. Cutaneous manifestations (urticaria) may also be associated with the presence of *Blastocystis* spp. [7]. Nevertheless, some investigators report no association between the presence of clinical manifestations and *Blastocystis* infection [8]. Hence, more information is required to properly assess the potential pathogenicity of this parasite.

Few studies have investigated the prevalence of *Blastocystis* infection in Spain, and most of them focused on children or symptomatic patients [9–11]. The aim of the present study was to describe the epidemiological and clinical characteristics of adult patients with *Blastocystis* infection and evaluate possible risk factors associated with the presence of clinical manifestations.

## Methods

A retrospective observational study was performed at the Vall d’Hebron University Hospital, a tertiary hospital included in the International Health Program of the Catalan Health Institute (PROSICS Barcelona, Spain). The study included all adult patients (≥18 years old) who attended the hospital from February 2009 to March 2014, had *Blastocystis* spp. in their stool and medical records available.

To establish the study cohort, a list of patients infected with *Blastocystis* sp. was compiled by examination of the Microbiology Department registry. Next, medical records were reviewed and for those patients with a medical record available, the following data was collected: epidemiological data (age, gender, country of origin, previous travels), immune status, clinical symptoms that could be related with *Blastocystis* sp. infection (gastrointestinal and cutaneous), detection of other intestinal parasites, treatment received and follow-up after treatment. *Blastocystis* sp. detection was performed by microscopic examination of stool samples following concentration using the Ritchie’s formalin-ether technique.

According to the geographical origin of patients and recent travels, patients were classified into four epidemiological risk groups: immigrants, travelers (patients born in Spain who have traveled outside the country in the last year before their *Blastocystis* sp. infection), “visiting friends and relatives” (VFR, immigrants living in Spain who have traveled to their country of origin in the last year before their *Blastocystis* sp. infection), and autochthonous infections (patients born in Spain with no history of recent travel outside the country).

To identify risk factors associated with the presence of symptoms, patients were classified into three groups: asymptomatic patients, symptomatic patients, and patients with symptoms that could be attributable to causes other than *Blastocystis* sp. infection.

In patients who received specific treatment for their *Blastocystis* sp. infection, microbiological and clinical follow-up information was collected. Regarding clinical response to therapy, patients were classified into three groups: those who experienced complete resolution of symptoms, those who reported an improvement in their symptoms but not complete resolution, and patients who experienced no clinical improvement following treatment.

Categorical data are presented as absolute numbers and proportions, and continuous variables are expressed as medians and ranges. The *χ* ^2^ test or Fisher’s exact test, when appropriate, was used to compare the distribution of categorical variables, and the Mann-Whitney U-test for continuous variables. To evaluate the seasonality of the infection, the two-sample test of proportions was performed between seasons. Results were considered statistically significant if the 2-tailed *P*-value was < 0.05. SPSS software for Windows (Version 19.0; SPSS Inc, Chicago, IL, USA) was used for statistical analyses.

## Results

**Fig. 1:**
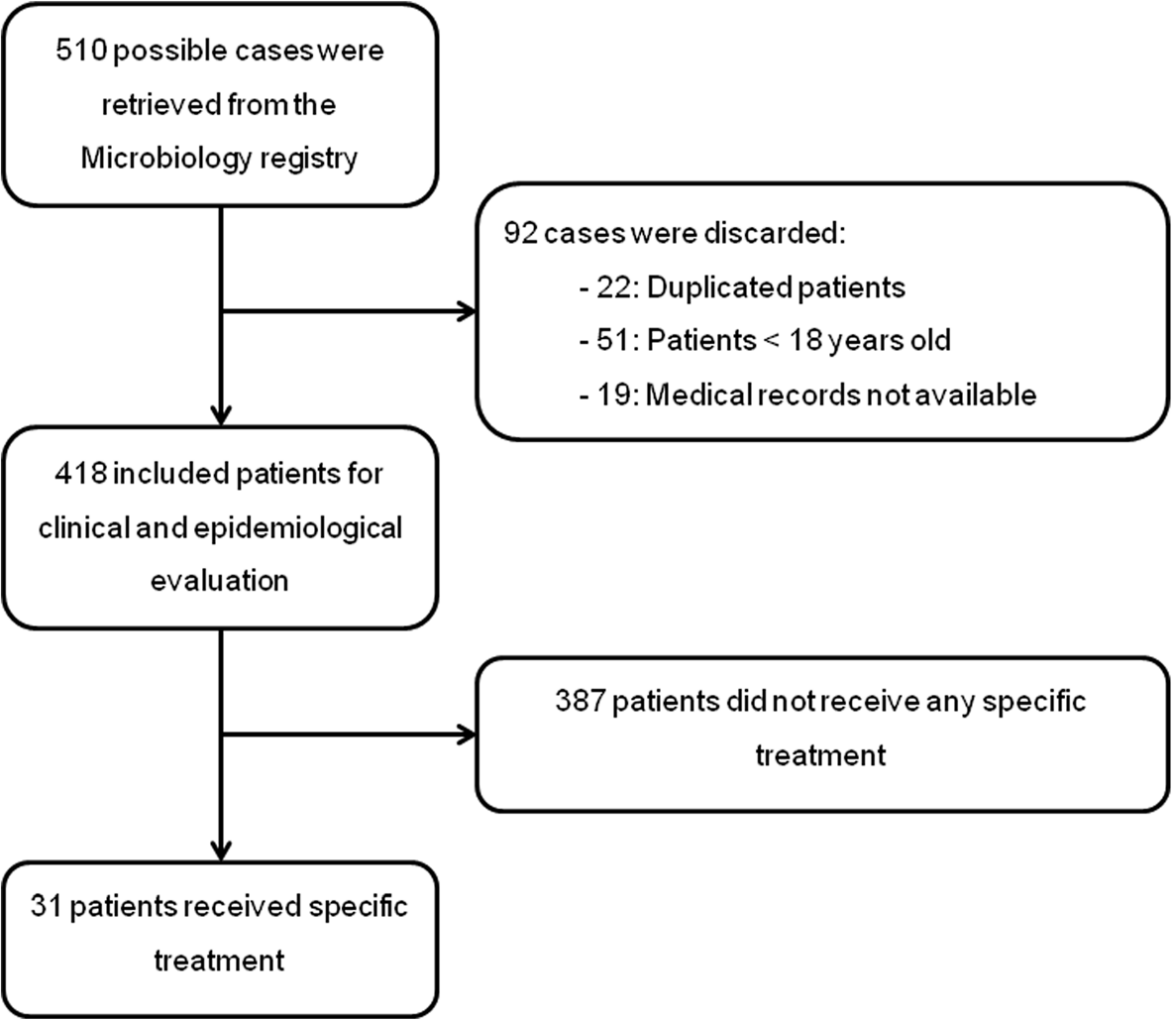
Flow diagram depicting study cohort size and selection

After consulting the Microbiology Department registry, 510 possible cases of *Blastocystis* sp. infection were identified. After discarding patients less than 18 years old, duplicated patients, and patients lacking complete medical records, 418 cases remained for inclusion in the study (see Fig. 1). The epidemiological characteristics of patients are summarized in Table 1. The median age of patients was 36 (18–86) years, and 236 (56.5 %) were men. Regarding immigration status, 263 (62.9 %) patients were immigrants with median time of residence in Spain of 60 (1–480) months, 26 (6.2 %) patients were travelers, 10 (2.4 %) patients were VFR, and 119 (28.5 %) cases were autochthonous. Most patients were diagnosed in spring, although this difference was not statistically significant when compared to *Blastocystis* sp. infections diagnosed in winter (32.3 *vs* 22.2 %, *Z* = 1.66, *P* = 0.08), summer (32.3 *vs* 22 %, *Z* = 1.69, *P* = 0.09), and autumn (32.3 *vs* 23.4 %, *Z* = 1.48, *P* = 0.13).

**Table 1:** Epidemiological characteristics of patients with *Blastocystis* sp. infection attended in Vall d’Hebron University Hospital, Barcelona (2009–2014). Data are reported as number (%) of patients or median (range)

Characteristics	Number of patients (*n* = 418)
Age, years	36 (18–86)
Gender, male	236 (56.5 %)
Origin of patients	
Spain	143 (34.2 %)
Latin America	149 (35.6 %)
Sub-Saharan Africa	98 (23.4 %)
Asia	14 (3.3 %)
North Africa	11 (2.6 %)
Other European countries	3 (0.7 %)
Epidemiological risk group	
Immigrants	263 (62.9 %)
Autochthonous	119 (28.5 %)
Travelers	26 (6.2 %)
Visiting friends and relatives	10 (2.4 %)

Sixty-six (15.8 %) patients were immunocompromised. Forty-three patients had HIV infection, with a median of CD4+ cell count of 428 (100–1,053) cells/mm^3^ at the time their *Blastocystis* sp. infection was diagnosed, 11 patients had a solid tumor or a haematological malignancy, and 12 patients had rheumatological, gastrointestinal or dermatological diseases requiring immunosuppressive treatment.

Regarding patient symptoms, 234 (56 %) patients were completely asymptomatic, 92 (22 %) patients had symptoms, and 92 (22 %) patients had symptoms that could be attributed to other causes. Information on the clinical characteristics of this cohort is available in Table 2. Among patients with other etiologies that could be responsible for their symptoms, ten had been diagnosed with intestinal neoplasm, 24 patients with other infectious diarrhea (*Clostridium difficile*, *Campylobacter jejuni*, *Salmonella enteritidis*, *Shigella disenteriae*, *Giardia duodenalis*), six patients with inflammatory bowel disease, and 11 patients with irritable bowel syndrome. Other intestinal parasites found in the study population are described in Table 3. From the 92 patients with symptoms not attributable to etiologies other than *Blastocystis* sp. infection, the duration of symptoms was < 1 month in 32 (34.8 %) patients, and > 1 month in 60 (65.2 %) patients. The most frequent symptoms were diarrhea (61 patients, 66.3 %) and abdominal pain (34 patients, 37 %), and 9 (9.8 %) patients had cutaneous manifestations including eczematous or urticarial dermatitis.

**Table 2:** Clinical characteristics of patients with *Blastocystis* sp. infection attended in Vall d’Hebron University Hospital, Barcelona (2009–2014)

Characteristics	Number of patients (%) (*n* = 418)
Presence of immunosuppression	66 (15.8)
HIV infection	43 (10.3)
Oncohaematological disease	11 (2.6)
Rheumatological disease	6 (1.4)
Other immunosuppressant conditions	6 (1.4)
Clinical groups according presence of symptoms	
Asymptomatic patients	234 (56)
Symptomatic patients	92 (22)
Symptomatic patients with other possible explanation	92 (22)
Symptoms among symptomatic patients (*n* = 92)^a^	
Duration of symptoms	
< 1 month	32 (34.8)
> 1 month	60 (65.2)
Diarrhea	61 (66.3)
Abdominal pain	34 (37)
Dyspepsia	16 (17.4)
Flatulence	16 (17.4)
Nausea/Vomiting	15 (16.3)
Abdominal distension	13 (14.1)
Pyrosis	4 (4.3)
Cutaneous manifestations	9 (9.8)

^a^Excluding symptomatic patients with other possible explanations

**Table 3:** Other intestinal parasites observed in stool samples of the study population

Parasites isolated	Number of patients (%) (*n* = 418)
Pathogenic parasites	
*Strongyloides stercoralis*	24 (5.7)
*Giardia duodenalis*	17 (4)
Hookworms	10 (2.4)
*Trichuris trichiura*	8 (1.9)
*Schistosoma mansoni*	3 (0.7)
*Schistosoma intercalatum*	2 (0.5)
*Hymenolepis nana*	2 (0.5)
*Taenia* sp.	2 (0.5)
*Ascaris lumbricoides*	1 (0.2)
Parasites of minor importance	
*Dientamoeba fragilis*	68 (16.2)
*Entamoeba histolytica/dispar*	3 (0.7)
Non pathogenic parasites	
*Endolimax nana*	66 (15.8)
*Entamoeba coli*	52 (12.4)
*Iodamoeba butschlii*	1 (0.2)
*Chilomastix mesnili*	1 (0.2)

When comparing the clinical and epidemiological characteristics of the asymptomatic and symptomatic patients with symptoms attributable to *Blastocystis* sp. alone, asymptomatic patients were younger (median age 34 years *vs* 42.5 years, *Z* = -3.896, *P* < 0.0001), included a larger proportion of migrants (89.7 *vs* 32.6 %, *χ* ^*2*^ = 110.998, *df* = 1, *P* < 0.0001), and included a greater proportion of immunocompromised patients (20.1 *vs* 5.4 %, *χ* ^*2*^ = 10.573, *df* = 1, *P* = 0.001) (Table 4).

**Table 4:** Comparison of clinical and epidemiological characteristics between symptomatic and asymptomatic patients with *Blastocystis* sp. infection attended in Vall d’Hebron University Hospital (2009–2014). Data are reported as number (%) of patients or median (range)

	Asymptomatic patients (*n* = 234)	Symptomatic patients^a^ (*n* = 92)	*P–*value
Age, years	34 (18–84)	42.5 (18–84)	< 0.0001
Gender, male	140 (59.8 %)	49 (53.3 %)	0.280
Foreign origin	210 (89.7 %)	30 (32.6 %)	< 0.0001
Immunosuppressant condition	47 (20.1 %)	5 (5.4 %)	0.001

^a^Excluding symptomatic patients for other reasons

Overall, 31 (7.4 %) symptomatic patients received specific treatment for *Blastocystis* sp. infection: 27 patients received 750 mg metronidazole three times a day for 10 days, two received 800 mg/160 mg cotrimoxazole two times a day for 7 days, and two patients received sequential treatment with metronidazole and cotrimoxazole (10 days of 750 mg metronidazole followed by 7 days of 800 mg/160 mg cotrimoxazole). The clinical response to treatment varied in this group. Five patients experienced complete resolution of symptoms and 12 reported some clinical improvement but not complete resolution of their illness. Eight patients described no clinical improvement following treatment, and information on the efficacy of treatment was not available for six patients. Therefore, more than 50 % of treated patients reported resolution or improvement of symptoms. Stool examination following treatment was performed for 12 patients. Microbiological cure was observed in seven patients, and five patients still had *Blastocystis* sp. in their stool. Six patients that experienced microbiological cure were treated with metronidazole, and one had received cotrimoxazole. Four of these patients reported improvement or resolution of symptoms and three patients reported no clinical improvement.

## Discussion

We retrospectively studied 418 adult patients with *Blastocystis* sp. infection who attended the Vall d’Hebron University Hospital. Most of them were young (median age of 36 years), and foreign-born (> 60 % of patients were born in a country different from Spain). Most of the patients were asymptomatic, but 22 % of them presented with symptoms that were most likely attributable to *Blastocystis* sp. infection given the absence of any other potential etiology.

Interest in *Blastocystis* sp. has significantly grown in recent years, due to its potential role as a human pathogen [3]. The prevalence of *Blastocystis* sp. infection varies depending on the study setting, reaching the highest percentages (68–100 %) among children from developing countries [12, 13]. Few studies have been performed in Spain, and most involve children with prevalences ranging from 5–20 % [9, 10]. In a previous study carried out by our group among foreign-born HIV infected adult patients, the prevalence of *Blastocystis* sp. infection was 17.3 % [14].

Seasonal distribution of *Blastocystis* sp. infection was reported previously in the United Kingdom, with higher incidences observed in summer [15]. In our study, although the number of cases was higher in spring, this was not statistically significant. A previous study performed in the same geographical area (Catalonia, Spain) also showed no seasonal differences [11].

In the present study, the most frequent symptoms observed in patients with illness that could only be attributable to *Blastocystis* sp. infection were diarrhea, abdominal pain, dyspepsia, flatulence and vomiting, similar to other studies [3]. While its pathogenicity has been questioned, *Blastocystis* sp. is now thought to cause gastrointestinal symptoms under some circumstances, sometimes of a severe nature [16, 17]. Although less common, cutaneous manifestations have been reported. In some patients acute or chronic urticaria has been observed, unexplained by other causes, with resolution of symptoms described following antiparasitic treatment [18, 19]. Nine patients in our study presented urticarial dermatitis without a reasonable cause, except for *Blastocystis* sp. infection. Some suggest that cutaneous symptoms may be caused by disruptions in immune homeostasis as the host produces an inflammatory response against the amoeboid forms of the parasite [20].

It has been suggested that gastrointestinal symptoms could be related with the ST of the parasite, but this remains uncertain [6]. ST-1 seems to be the most pathogenic one, while ST-3, although being the most common ST in humans, is less frequently associated with symptoms [6, 21]. Parasite loads as observed by light microscopy may also be related to the presence or absence of symptoms, with symptoms occurring more frequently when more than five parasites per high-power field (400×) are observed [22].

*Blastocystis* sp. infection may cause symptoms similar to those attributed to irritable bowel syndrome (IBS). Several studies have shown the possible link between *Blastocystis* sp. infection and IBS, and *Blastocystis* sp. infections are more prevalent among patients with IBS when compared to control groups [23, 24]. While this is the case, links between IBS and any particular *Blastocystis* sp. ST have not been observed [25, 26]. A study by Nourrisson et al. revealed that patients with IBS and *Blastocystis* sp. infection had significantly lower levels of beneficial gut bacteria including *Bifidobacterium* spp. and *Faecalibacterium prausnitzii*, supporting that *Blastocystis* sp. might be linked to the pathophysiology of IBS and intestinal flora imbalance [27]. In our cohort of *Blastocystis* sp. infected patients, 11 had been diagnosed with IBS, although only one patient received antiparasitic treatment and clinical improvement was not observed. Nevertheless, this association between IBS and *Blastocystis* sp. requires further investigation given the conflicting reports on this subject [28].

In the present study, the asymptomatic patient group had a higher proportion of foreigners and immunosuppressed patients compared to the symptomatic patient group. These differences could be linked to biases related to the types of patients that are screened for bowel parasites, because fecal microscopic examination for intestinal parasitosis is routinely performed in our hospital on immigrants, particularly those who are immunocompromised regardless of whether they are symptomatic or asymptomatic. Information on the pathogenicity of *Blastocystis* sp. in immunocompromised patients is unavailable and only limited information on its prevalence in immunosuppressed cohorts has been reported, mostly for HIV infected patients [14, 29, 30].

Only 31 symptomatic patients received antiparasitic treatment for *Blastocystis* sp. infection in our study, making it difficult to speculate on the usefulness of treatment for *Blastocystis* sp. infections. Given the controversy surrounding the pathogenic potential of this parasite, physicians are still uncertain as to the value of treating *Blastocystis* sp. infections. Metronidazole is the most frequently administered drug for *Blastocystis* sp. infection, though its efficacy is highly variable [3]. Other studies have shown the potential benefits of other drugs, such as nitazoxanide, paromomycin or trimethoprim-sulfamethoxazole [31, 32]. Despite all the controversies, the treatment success rate achieved in this study (more than 50 %), suggest that treatment should be considered in patients with chronic symptoms in the absence of other reasonable causes.

This study has some limitations derived from its retrospective nature. Among these limitations is the fact that information retrieved from medical records is not always complete, different physicians were involved in the diagnosis, and differences exist in the way patients were assessed. Diagnosis was based on the microscopic examination of stool samples, lacking information relating to the STs or parasite burden. Moreover, the formalin-ether technique has reduced sensitivity in detecting *Blastocystis* sp. compared to other techniques which may have reduced its recovery in this cohort [15]. Finally, the small sample size and the varied responses to chemotherapy make it difficult to assess the efficacy of antiprotozoal treatment in this cohort.

## Conclusions

In summary, 418 *Blastocystis* sp. infected patients were diagnosed in Vall d’Hebron University Hospital over a 5-year period, most of them were foreign-born patients (> 60 %). Although the vast majority of patients were asymptomatic, 22 % of patients had gastrointestinal symptoms or cutaneous manifestations in the absence of other potential etiologies. Despite the scarcity of information available, and given the safety of antiparasitic treatment, the results of the present study suggest that antiprotozoal treatment should be considered in patients infected with *Blastocystis* sp. experiencing chronic symptoms. The present study also suggests that *Blastocystis* sp. are pathogenic at least under some circumstances, and should be considered a cause of illness in the absence of any other etiology.

## Abbreviations

IBS: Irritable bowel syndrome
SSU rRNA: Small subunit rRNA
ST: Subtype
VFR: Visiting friends and relatives
